# Case report: Antenatal MRI diagnosis of cloacal dysgenesis syndrome

**DOI:** 10.4103/0971-3026.63041

**Published:** 2010-05

**Authors:** P Gupta, S Kumar, Raju Sharma, A Gadodia

**Affiliations:** Department of Obstetrics and Gynecology, All India Institute of Medical Sciences, New Delhi, India; 1 Department of Radio-Diagnosis, All India Institute of Medical Sciences, New Delhi, India

**Keywords:** Cloacal dysgenesis, fetus, MRI, prenatal diagnosis

## Abstract

Cloacal dysgenesis sequence (CDS) is a lethal malformation with a highly variable presentation. CDS is characterized by direct communication between the gastrointestinal, urinary, and genital structures, resulting in a single perineal opening. Prenatal diagnosis of a cloacal anomaly is often difficult because of the highly variable imaging features. Here, we report a case in which a diagnosis of CDS was made with fetal MRI on the basis of a meconium-containing, bilobed, abdominopelvic cystic mass communicating with the ureters and the colon.

## Introduction

Cloacal malformation is a rare anomaly with a highly variable presentation. The incidence is about 1 in 50,000 live births.[1] This complex malformation is a rare cause of fetal obstructive uropathy.[2] Cloacal anomalies represent the persistence of an early embryonic development in which the urinary, genital, and gastrointestinal tracts remain confluent and communicate with the exterior through a single perineal opening.[3,4]

The prenatal diagnosis of a cloacal malformation may allow better planning of pre- and perinatal care, but is difficult because of the highly variable imaging features, depending on the type of malformation and the gestational age.[1–3] Fetal MRI has been recognized as a complementary tool to USG in the diagnosis of genitourinary and gastrointestinal disorders.[5,6] We report a case of cloacal dysgenesis sequence (CDS) that was diagnosed as obstructive uropathy on USG. A subsequent fetal MRI clearly delineated a cloacal structure and identified the connection and continuity of the cystic mass with the urinary and intestinal tracts.

## Case Report

**Figure 1:**
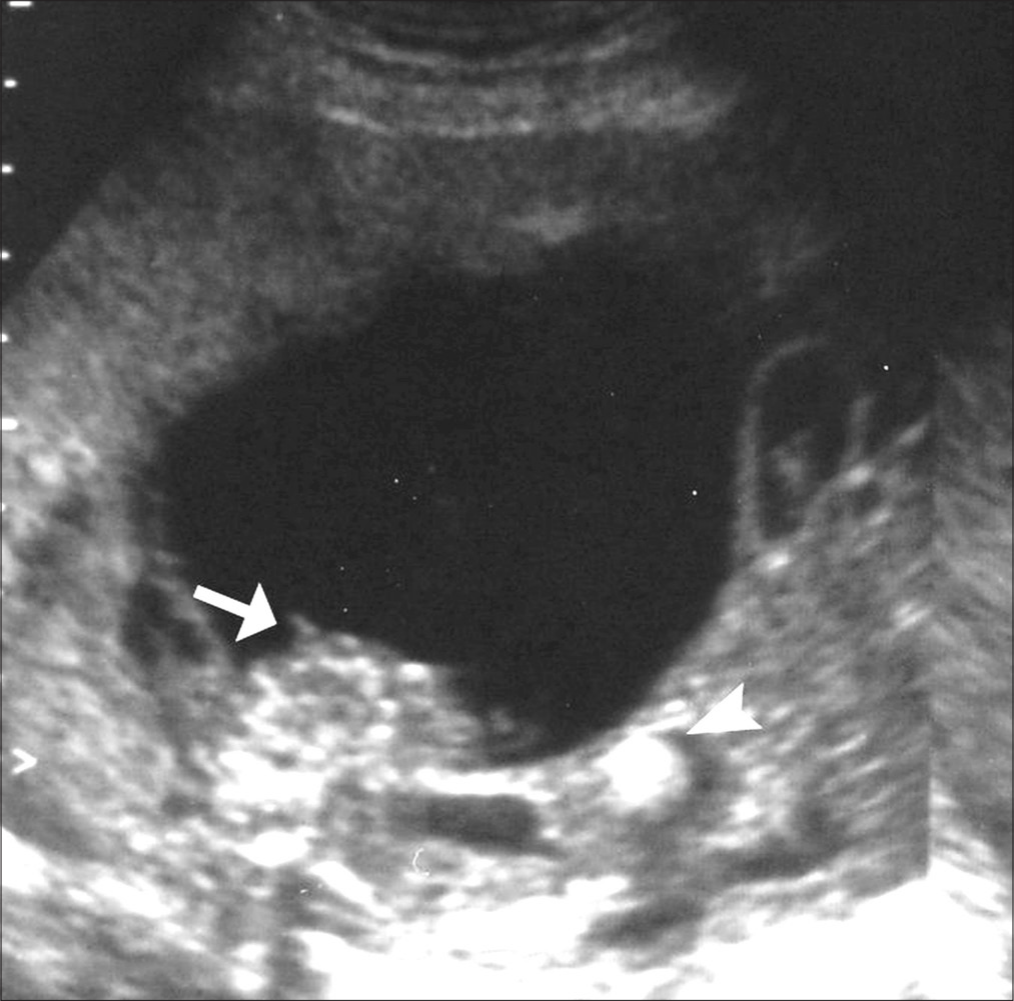
Axial USG shows a large, cystic, abdominopelvic mass containing debris (arrow). The bladder was not seen separate from the mass lesion. The bowel loops were dilated and foci of calcified meconium were seen in the lumen (arrowhead)

A 29-year-old primigravida underwent routine antenatal USG at another institution at 28 weeks gestation. This revealed a large abdominal cystic mass and oligohydramnios. Her history was otherwise unremarkable. She had not been exposed to any teratogenic agent during pregnancy. Repeat USG at our institution showed a single live fetus with a vertex presentation and a biparietal diameter and femur length corresponding to 28 weeks gestation. A 10 × 15 cm hypoechoic, abdominopelvic cystic mass, containing debris was seen. The bladder was not seen separate from the mass lesion. The mass was pushing the diaphragm, causing lung hypoplasia. The right kidney was enlarged and hydronephrotic, while the left kidney was not visualized separately. The bowel loops were dilated and multiple foci of calcified meconium were seen in the lumen [Figure 1]. The amniotic fluid was severely reduced. There were no signs of generalized hydrops or pleural effusion. Fetal sex could not be determined. A possibility of fetal obstructive uropathy was considered based on the presence of the midline pelvic structure, the hydronephrotic kidney, and the severe oligohydramnios. Cloacal dysgenesis was also suspected on the basis of the echogenic calcification in the colonic lumen as well as the cystic mass lesion. Fetal karyotyping and other invasive procedures were refused by the mother.

**Figure 2(A-F):**
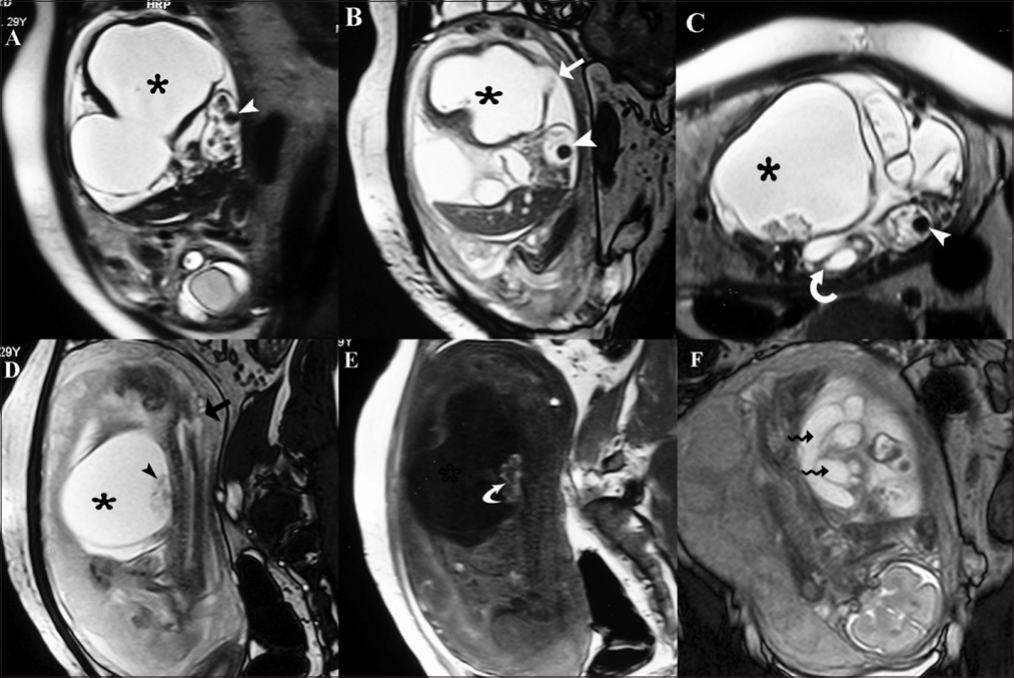
Antenatal MRI. Sagittal HASTE (A), Tru-FISP (B), and axial HASTE (C) images show a bilobed, cystic, abdominopelvic mass (asterisk) communicating with the dilated bowel (white arrow). Also, note the presence of enterolithiasis in the dilated bowel loop (white arrowhead in A-C). The bladder is not seen separate from the cystic mass. The right kidney is hydronephrotic (curved arrow in C). Sagittal Tru-FISP (D) and T1W (E) images show that the cystic lesion (asterisk) shows T2 hyperintensity and T1 hypointensity. Subtle T1 hyperintense foci suggestive of meconium are seen within the cystic mass (curved arrow in E). Also, note the presence of a lumbar meningocele (black arrow in D). In addition, debris is seen involving the cystic structure (black arrowhead in D). A sagittal Tru-FISP (F) image demonstrates the presence of a double ureter (wavy arrow) till the lower end; the ureters drain into the cystic lesion

MRI (Sonata, Siemens; Erlangen, Germany) was performed to further evaluate the fetus using half-Fourier single-shot turbo spin-echo (HASTE), true fast imaging with steady-state precession (Tru-FISP), and gradient echo T1W sequences. MRI revealed a large, bilobed, abdominopelvic cystic mass [Figure 2A]. Its wall was thick anteriorly and thin posteriorly. The bladder and rectum were not seen separately in the pelvis. The colon was dilated and ended blindly in the abdominal cyst [Figure 2A–C]. The lesion was hypointense on T1W images, with foci of hyperintense signal on T1W images, suggestive of meconium [Figure 2E]. Multiple, hypointense, round foci were seen on T2W images in the lumen of the dilated colon, suggestive of enterolithiasis [Figure 2A–C]. The right kidney was hydronephrotic and showed double ureters, both opening on the anterolateral aspect of the cystic lesion [Figure 2C,F]. The left kidney was not visualized in the renal fossa. A small lumbar meningomyelocele was also noted [Figure 2D]. The perineum was smooth and the external genitalia were not seen, indicating a female fetus.

**Figure 3:**
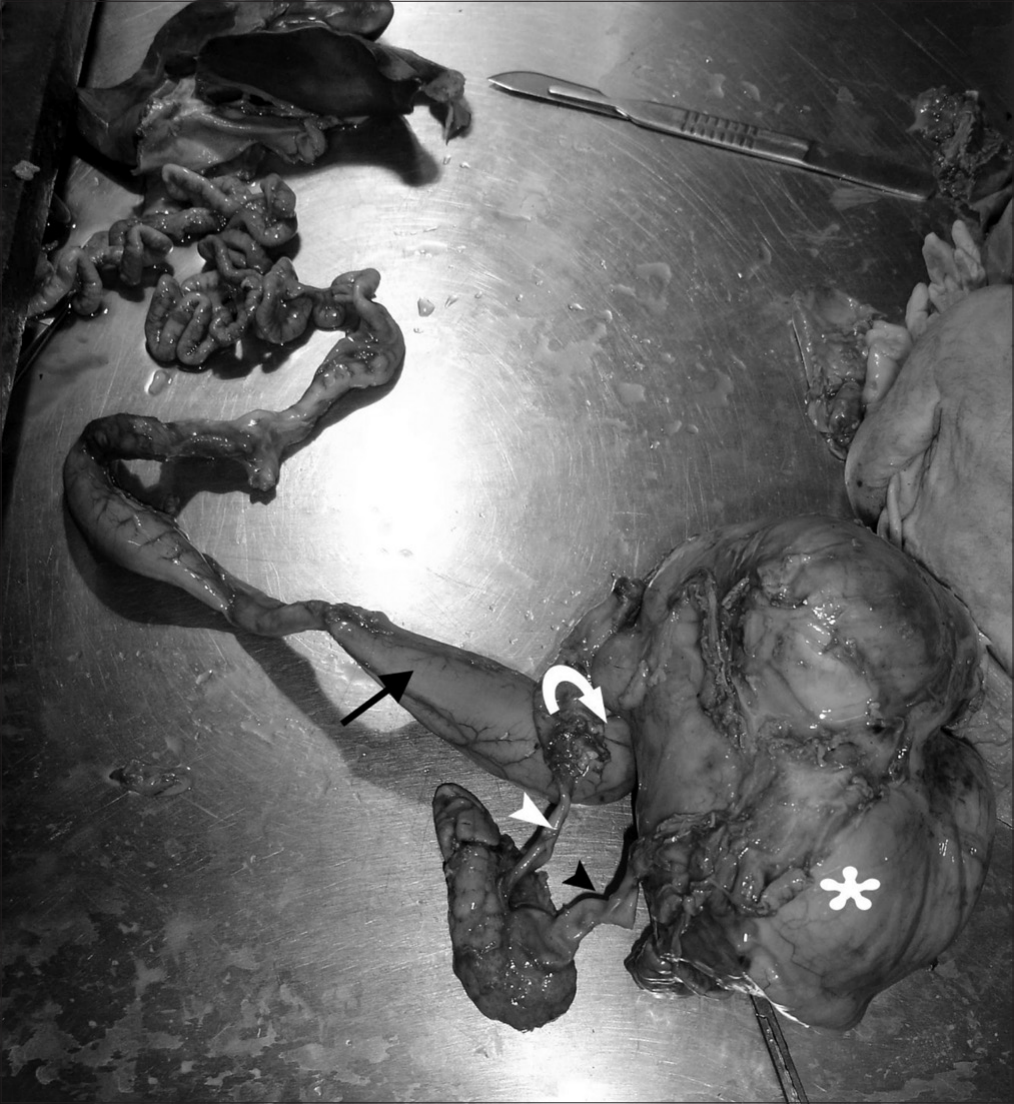
Postmortem examination reveals a large cystic mass lesion (asterisk), communicating with the dilated colon (arrow). The small bowel loops were normal. The right kidney is hydronephrotic, with double ureters (arrowhead) draining into the cystic mass. The left kidney was not seen

A diagnosis of CDS with left renal agenesis and lumbar meningomyelocele was made. Poor prognostic features included severe oligohydroamnios, pulmonary hypoplasia, and enterolithiasis. The parents were informed of the poor chances of survival and the patient was followed up with serial USG every 2 weeks. Intrauterine demise of the fetus was detected at 34 weeks gestation. Labor was induced with 50 μg misoprostol. A fetus weighing 1.9 kg, with an extremely protuberant abdomen, imperforate anus, and ambiguous genitalia was delivered. Gross examination revealed a smooth perineum, without patent urethral, vaginal, and anal openings; enlarged wrinkled clitoral-like structure; and talipes equinovarus. Postmortem examination revealed a single enlarged right kidney, with double ureters draining into a large pelvic cystic lesion [Figure 3]. The large bowel was also opening into the cloaca, and calcified meconium was seen in the colon as well as the cloaca. The cloaca showed transitional lining, with musculature resembling that of the urinary bladder. The left kidney was absent and there was pulmonary hypoplasia. The stomach and small intestine were normal. Chromosomal analysis showed a normal female karyotype.

## Discussion

Cloacal anomalies can have varied manifestations, ranging from a persistent cloaca to complete breakdown of the cloacal membrane with exstrophy, failure of fusion of the genital tubercles, and omphalocele.[3,4] CDS is considered when severe anorectal atresia occurs in association with a common cavity for the hindgut and urogenital sinus. The primary malformation includes a smooth perineum, with the absence of labioscrotal development and absence of anal, genital, and urinary orifices. In CDS, the colon often ends blindly at the posterior aspect of the urinary bladder. The mean gestational age at diagnosis is 27 weeks (19–33 weeks), but many cases are diagnosed only in the third trimester or after birth.[7] Inability to demonstrate communication between the genitourinary and gastrointestinal tracts with USG makes prenatal diagnosis of cloacal anomalies challenging.[8]

The cloaca forms from the developing tail fold at 3 weeks through the confluence of the allantois and the hindgut.[3,4] The cloaca is subdivided into the urogenital sinus anteriorly and the hindgut posteriorly between the 5th and 7th weeks by the craniocaudal growth of the urorectal septum. Failure of the cloaca to subdivide leads to the persistence of the cloaca; arrest can occur at any point, leading to a wide spectrum of cloacal dysgenesis, clinically. The extent of the lesion depends on the degree of the developmental defect in the early mesoderm.[3,4] Initially, it was believed that a persistent cloaca occurs only in females;[9] however, recent reports have described CDS in males also.[2,10]

Antenatal USG findings include transient fetal ascites, a bilobed debris-filled, abdominopelvic cystic structure, poorly or nonvisualized bladder, oligohydramnios, cystic or dysplastic kidneys, and hydronephrosis.[11,12] A characteristic feature that should be sought for is folding of the dilated bladder, which represents the margin of the communication between the bladder and (usually) the female genital tract.[13] A bewildering range of anomalies is reported to occur, which includes ureteral ectopia, bladder and urtheral duplications, uterine and vaginal duplications and atresia, renal agenesis, horseshoe kidney, omphalocele, tracheoesophageal fistulas, duodenal atresias, patent ductus arteriosus, myelomeningoceles, sacral agenesis, tethered spinal cord, and vertebral segmentation anomalies.[1–13] Another possible clue to the prenatal diagnosis of CDS is the presence of intraluminal calcifications in the dilated bowel loop. Intraluminal calcification of meconium appears to result from the mixing of stagnant urine and meconium *in utero*, and its presence should suggest the presence of a fistula between the urinary and the gastrointestinal tracts.[2,10] The prognosis for a fetus with CDS is extremely poor. Poor prognostic features include severe oligohydramnios, pulmonary hypoplasia, and enterolithiasis.[1,10] Oligohydramnios may also develop as a consequence of urinary tract obstruction, and may lead to pulmonary hypoplasia if it occurs before 24 weeks of gestation. The differential diagnosis of this lesion includes bowel atresia, megacystis-microcolon-intestinal hypoperistalsis syndrome, and obstructive uropathy.[2]

Fetal MRI has recently been used in the diagnosis of cloacal anomalies.[1,8,10,14] MRI provides high-quality fetal images, regardless of maternal or fetal position or the presence of oligohydramnios. Fetal MRI helps in better anatomical localization of the cloacal anomalies by delineating the communication between the cyst, gastrointestinal tract, and ureter. In addition, MRI is of supplemental value to USG because of improved tissue characterization. MRI is more sensitive than USG for detecting the presence of meconium. Meconium exhibits intermediate or low signal intensity on T2W images and a high signal intensity on T1W images because of its high protein and mineral content.[5,6] T1W images may add additional information to that obtained from T2W images for the diagnosis of fetal gastrointestinal abnormalities because meconium is more apparent on T1W than on T2W images. In the present case, MRI showed both the hindgut and the ureters opening into the pelvic cyst as well as the presence of meconium in the cyst, thus confirming the diagnosis of CDS.

In our case, CDS was diagnosed prenatally on the basis of the presence of a bilobed, cystic, abdominopelvic mass containing meconium and having showing communication with the ureter and the colon. MRI not only defined the bilobed pelvic cyst but also demonstrated the possible communication with the bowel and the ureter, which helped us make a more precise diagnosis prenatally.

In summary, CDS should be considered in the differential diagnosis of a midline, cystic, abdominopelvic mass in a female fetus. Prenatal USG diagnosis of this entity is difficult. Antenatal MRI plays an important role in making a precise prenatal diagnosis by demonstrating the communication between the genitourinary and gastrointestinal tracts. MRI is also more sensitive than USG for detecting the presence of meconium.
